# Safety and efficacy of trifluridine/tipiracil in previously treated metastatic colorectal cancer: final results from the phase IIIb single-arm PRECONNECT study by duration of therapy

**DOI:** 10.1186/s12885-022-10489-4

**Published:** 2023-01-27

**Authors:** Julien Taieb, Timothy Price, Loïck Vidot, Bénédicte Chevallier, Lucjan Wyrwicz, Jean-Baptiste Bachet

**Affiliations:** 1 Department of Gastroenterology and Digestive Oncology, European Hospital Group Georges-Pompidou, Paris, France; 2 Department of Medical Oncology, The Queen Elizabeth Hospital and University of Adelaide, Woodville South, South Australia Australia; 3 Servier, Suresnes, Paris, France; 4 Department of Oncology and Radiotherapy, Maria Skłodowska Curie Memorial Cancer Centre and Institute of Oncology, Warsaw National Cancer Research Institute, Warsaw, Poland; 5 Department of Hepatogastroenterology, University Hospital Pitie Salpetriere, Paris, France; 6 Sorbonne University, Paris, France

## Abstract

**Background:**

PRECONNECT was an international, phase IIIb trial evaluating the safety and efficacy of trifluridine/tipiracil (FTD/TPI) for metastatic colorectal cancer (mCRC).

**Methods:**

Patients with mCRC received FTD/TPI 35 mg/m^2^ twice-daily on days 1–5 and 8–12 of each 28-day cycle for third- or later-line treatment. Primary endpoint: safety and time to deterioration of Eastern Cooperative Oncology Group performance status [ECOG PS] to ≥2). Secondary endpoints included progression-free survival (PFS). Potential prognostic factors for PFS were explored.

**Results:**

Of 914 patients, 69% completed 0–3, 24% completed 4–7, and 7% completed ≥8 cycles of FTD/TPI. Drug-related grade ≥ 3 adverse events included neutropenia (38.1%), anaemia (7.2%) and asthenia (3.4%). Median [95% CI] time to ECOG PS deterioration was 8.7 [8.1-not calculable] months and increased with duration of treatment (DoT). Median PFS was 2.8 [2.7–3.0] months and increased with duration of treatment DoT. Prognostic factors associated with longer PFS included time since diagnosis of first metastasis, number of metastatic sites, baseline ECOG PS, presence/absence of liver metastasis or previous regorafenib treatment, and laboratory variables.

**Conclusions:**

No new safety concerns for FTD/TPI were identified and PFS increased with DoT. These data provide confidence for the use of FTD/TPI, including the use of multiple cycles, in routine practice.

**Trial registration:**

EudraCT Number: 2016-002311-18; registered 19/09/2016.

https://clinicaltrials.gov/ct2/show/NCT03306394; registered 11/10/2017.

**Supplementary Information:**

The online version contains supplementary material available at 10.1186/s12885-022-10489-4.

## Introduction

Globally, colorectal cancer (CRC) is ranked as the third most common cancer and the second leading cause of cancer-related death, accounting for approximately 10% of all new cases of cancer and 10% of all deaths from cancer in 2020 [1]. Survival of patients with CRC varies depending on the stage at diagnosis [2], and primary and secondary treatments for unresectable metastatic CRC (mCRC) are based on combinations of cytotoxic chemotherapy, biological therapy such as antibodies and targeting cellular growth factors, and immunotherapy [3]. Owing to improvements in first- and second-line treatments, a growing number of patients with mCRC are candidates to receive a treatment in third-line or beyond.

Trifluridine/tipiracil (FTD/TPI), an oral cytotoxic chemotherapy consisting of the thymidine analogue trifluridine and the thymidine phosphorylase inhibitor tipiracil hydrochloride, is registered in many countries worldwide for the management of patients with mCRC who have progressed on standard therapies. FTD/TPI is indicated as monotherapy for the treatment of adults with mCRC who have been previously treated with, or are not considered candidates for, available therapies (including fluoropyrimidine-, oxaliplatin- and irinotecan-based chemotherapies, anti-VEGF agents, and anti-EGFR agents) [4].

In the pivotal RECOURSE study, FTD/TPI significantly improved overall survival (OS) and progression-free survival (PFS) compared with placebo [5]. Benefits of FTD/TPI were observed irrespective of age, geography or KRAS (v-Kiras2 Kirsten rat sarcoma 2 viral oncogene homologue) status [6]. Exploratory analysis of the RECOURSE study data suggested possible positive prognostic factors for PFS included < 3 metastatic sites when FTD/TPI treatment was initiated and ≥ 18 months from diagnosis of metastatic disease to inclusion. Patients with these disease characteristics had a median PFS of 3.3 months compared with 1.9 months in other patients in RECOURSE (*p* < 0.0001) [7].

Studies of the real-world use of FTD/TPI have suggested that carcinoembryonic antigen (CEA) < 200, neutrophil-to-lymphocyte ratio (NLR) < 5 and the development of grade 3 neutropenia may predict a favourable efficacy of FTD/TPI [8], while the number of previous lines of therapy was prognostic for OS and PFS in other studies [9]. However, a real-world study in Spain highlighted the difference between prognostic factors detected in different publications and suggested that the prognostic groups defined in RECOURSE did not apply to routine practice [10].

Further information is needed on factors that may influence the efficacy of FTD/TPI in mCRC, and therefore, the PRECONNECT study was initiated as an international, multicentre, open-label, phase IIIb trial, in which eligible adults with mCRC were provided access to FTD/TPI in clinical practice with the aim to further assess safety, clinical use, and quality of life (QoL) as measured by patient reported outcomes. PRECONNECT is the largest cohort of patients to date that have received FTD/TPI in clinical practice (*N* = 914), it included a multinational cohort (16 countries), and provides more detailed information on patients receiving multiple cycles of FTD/TPI than has been observed to date. Interim data from PRECONNECT were previously reported [11], and here we present final results from the PRECONNECT study according to the duration of treatment (DoT) and with an assessment of prognostic factors.

## Methods

### Study design

This was an international, multicentre, open-label, single-arm, phase IIIb trial (EudraCT Number: 2016-002311-18; registered 19/09/2016; https://clinicaltrials.gov/ct2/show/NCT03306394; registered 11/10/2017) in adults with pre-treated, histologically confirmed adenocarcinoma of the colon or rectum and with metastatic lesions. The primary objective was to collect additional safety data on FTD/TPI (incidence of adverse events and time to first ECOG PS deterioration) and secondary objectives included assessment of PFS and QoL.

### Patients

Patients were enrolled in 16 countries (Australia, Belgium, Brazil, Bulgaria, Croatia, France, Ireland, Italy, Panama, Poland, Portugal, Romania, Slovakia, Slovenia, Turkey, and Ukraine) and treated between October 2016 and November 2020. Eligible patients with mCRC were refractory to, or not candidates for, standard chemotherapies and had received ≥2 previous regimens of standard chemotherapies for mCRC (including fluoropyrimidines, irinotecan, oxaliplatin, an anti-vascular endothelial growth factor monoclonal antibody and ≥ 1 epidermal growth factor receptor monoclonal antibodies for RAS wild-type tumours), an Eastern Cooperative Oncology Group Performance Status (ECOG PS) of 0 or 1 during the screening period, and adequate renal (creatinine clearance ≥60 ml/min assessed with the Cockcroft-Gault formula), hepatic, cardiac and bone marrow function.

Exclusion criteria were: pregnancy, breast feeding or likely to become pregnant during the study; participation in another interventional study within 4 weeks of entering PRECONNECT; previously receiving or hypersensitive to FTD/TPI or excipients; had other active malignancies, brain metastasis, active infection, ascites, pulmonary fibrosis or uncontrolled diabetes; had major surgery within 4 weeks, and anticancer therapy within 3 weeks or bevacizumab treatment within 4 weeks, radiation therapy within 4 weeks; or any unresolved grade 2 or higher toxicity attributed to previous therapy.

### Treatment

Patients received oral FTD/TPI 35 mg/m^2^ twice daily (after morning and evening meals) on days 1–5 and 8–12 of each 28-day cycle. Delays, interruptions, or adjustments (in 5 mg/m^2^ reductions to a minimum of 20 mg/m^2^ twice daily) to the dose were based on individual safety and tolerability. Dose escalation was not permitted. Patients discontinued treatment if they met ≥1 of the following criteria: disease progression, unacceptable toxicity, withdrawal of consent, physician decision, pregnancy or major protocol deviation (defined as ‘a deviation that interferes with the study evaluations and/or jeopardises patient’s safety’). In addition, in any country where FTD/TPI became commercially available (i.e., market authorization or reimbursement was granted by the relevant authority in the country) the patients receiving FTD/TPI were withdrawn from the PRECONNECT trial and continued treatment outside the study; moreover, no further patient recruitment took place after commercial availability.

### Assessments and endpoints

Patients were assessed at baseline (within 7 days of the first FTD/TPI dose), and on the first day of each 28-day cycle of FTD/TPI treatment. Additional unscheduled assessments could be made according to the investigators’ assessment (e.g., for tumour and adverse event evaluation).

The primary endpoint was safety, assessed by the incidence of treatment-emergent adverse events (TEAEs), graded according to the National Institute of Health Common Terminology Criteria for Adverse Events version 4.03, from baseline through to the end-of-treatment visit, and by time to first ECOG PS deterioration [12]. Time to ECOG PS deterioration was defined as the time from the first dose of FTD/TPI until the first deterioration of ECOG PS from 0 or 1 at baseline to ≥2 post-baseline (or death without previous deterioration of ECOG PS to ≥2). If baseline ECOG PS was missing, an ECOG PS of 0 or 1 was assumed for statistical analyses.

Secondary endpoints included PFS, response rate (objective response rate [ORR] and disease control rate [DCR]), and QoL. PFS was defined as the time from the first intake of FTD/TPI until the date of investigator-assessed disease progression or death from any cause. ORR was defined as the proportion of patients achieving a complete response (CR) or partial response (PR), and DCR was defined as the proportion of patients achieving a CR, PR, or stable disease (SD). The assessment of response rates was by RECIST V1.1. The withdrawal criteria mentioned above were not compatible with an assessment of OS.

QoL was assessed using the European Organization for Research and Treatment of Cancer Quality of Life (EORTC QLQ-C30) health questionnaire (completed by the patients) [13]. Changes in QoL were considered clinically relevant if there was a ≥ 10-point change from baseline for the EORTC QLQ-C30 Global Health Status (GHS) score [14].

The number of 15 or 20 mg tablets of FTD/TPI dispensed per cycle, and the number of tablets returned and/or estimated to have been taken, was recorded. This information was used to estimate the administered dose and dose intensity.

In this analysis of PRECONNECT, data are presented for all patients that received ≥1 dose of FTD/TPI and in three subgroups according to duration of FTD/TPI treatment (i.e., 0–3, 4–7, or ≥ 8 cycles). Exploratory analyses were undertaken to examine potential prognostic factors for PFS, risk factors for neutropenia, and the association between neutropenia and PFS.

### Statistical methods

Descriptive statistics were used to summarise baseline patient and clinical characteristics, TEAEs, ECOG PS, and QoL. Median and 95% confidence intervals (CIs) were reported for survival analyses and were estimated using the Kaplan-Meier method. For the PFS analysis, patients without disease progression or death before or at the last visit (including patients withdrawn for commercial availability), were censored at the date of the last evaluable tumour assessment. For the time to ECOG PS deterioration analysis, patients not reaching an ECOG PS of ≥2 and not dead, were censored at the last recorded ECOG PS assessment.

A Cox proportional hazards model with time-dependent variable was used to explore the association between neutropenia and PFS. A Cox proportional hazards model including a stepwise selection process (including/retaining factors significant at the 10% level; Wald chi-square test) was used to explore potential prognostic factors for PFS. Factors considered for inclusion in the model were: time since diagnosis of the 1st metastasis (< 18 versus ≥18 months); number of metastatic sites (1–2 versus ≥3); ECOG PS (0 versus 1); presence of liver metastases (yes versus no); presence of lung metastases (yes versus no); presence of lymph node metastases (yes versus no); BMI group (< 18.5 versus ≥18.5; < 25 versus ≥25; < 30 versus ≥30); RAS status (wild versus mutant); side of primary tumour site (right versus left); gender (male versus female); age group (< 70 years versus ≥70 years); previous treatment with an anti-VEGF (yes versus no); previous treatment with regorafenib (yes versus no); and baseline haemoglobin (< 110 versus ≥110 g/l), white blood cell count (< 10 versus ≥10 × 10^9^/l), platelet count (< 400 versus ≥400 × 10^9^/l), neutrophil:lymphocyte ratio (NLR) (<3 versus ≥3; < 5 versus ≥5), albumin (< 35 versus ≥35 g/l), high total bilirubin (grade 0 versus grade ≥ 1), high aspartate aminotransferase (AST) (grade 0 versus grade = 1), high alanine aminotransferase (ALT) (grade 0 versus grade = 1) or high alkaline phosphatase (grade 0 versus grade ≥ 1). The final model was re-run to estimate hazard ratios and their 95% CI based on all patients with complete data on prognosis factors, including patients withdrawn for commercial availability.

A logistic regression model including a stepwise selection process was used to explore risk factors for neutropenia, with the same factors as for PFS described above with the addition of number of previous lines of treatment (≤2 versus > 2).

All statistical analyses were performed using SAS (V.9.2) software under the responsibility of Global Biostatistics of the sponsor (I.R.I.S).

## Results

Overall, 916 adult patients with mCRC were recruited in 16 countries. Of these, 914 patients received ≥1 dose of FTD/TPI; 632 (69%) completed 0–3 cycles, 219 (24%) completed 4–7 cycles, and 63 (7%) completed ≥8 cycles of FTD/TPI. Mean treatment duration was 3.6 months (SD 2.7; range 0.2–31.1 months). Most patients received a FTD/TPI relative dose intensity of 80–100%, and relative dose intensity decreased with increasing DoT (Supplementary Table 1). Progressive disease (80%) and commercial availability of FTD/TPI (10%) were the most frequent reasons for withdrawal.

At baseline, patients had a median age of 62 years, 59% were male, 52% had a mutant RAS tumour status, and 49% had an ECOG PS of 0 (Table 1). Although the significance or relevance are not clear, a numerically greater proportion of patients completing 4–7 or ≥ 8 cycles of FTD/TPI had a baseline ECOG PS of 0, and a numerically smaller proportion had a RAS mutation, and had a longer time from first metastasis to first FTD/TPI intake than patients completing 0–3 cycles (Table 1).

**Table 1:** Baseline characteristics of the whole population in PRECONNECT and by duration of FTD/TPI treatment

Characteristic^a^	All	0–3 cycles	4–7 cycles	≥8 cycles
	*N*=914	*n*=632	*n*=219	*n*=63
Age, years, median (min, max)	62 (24, 89)	62 (24, 89)	63 (30, 82)	65 (34, 83)
Age group				
<70 years	706 (77)	489 (77)	168 (77)	49 (78)
≥70 years	208 (23)	143 (23)	51 (23)	14 (22)
Gender				
Female	373 (41)	265 (42)	84 (38)	24 (38)
Male	541 (59)	367 (58)	135 (62)	39 (62)
Body mass index, kg/m^2^	*N*=893	*N*=615	*N*=215	*N*=63
<18.5	48 (5)	41 (7)	7 (3)	0 (0)
≥18.5	845 (95)	574 (93)	208 (97)	63 (100)
<25	451 (51)	342 (56)	85 (40)	24 (38)
≥25	442 (50)	273 (44)	130 (60)	39 (62)
<30	738 (83)	527 (86)	168 (78)	43 (68)
≥30	155 (17)	88 (14)	47 (22)	20 (32)
ECOG PS status				
All patients^b^	887	608	216	63
0	435 (49)	271 (45)	129 (60)	35 (56)
1	450 (51)	335 (55)	87 (40)	28 (44)
≥2	2 (0)	2 (0)		
Haematology				
Haemoglobin	*N*=872	*N*=598	*N*=212	*N*=62
<110 g/l	162 (19)	137 (23)	21 (10)	4 (6)
≥110 g/l	710 (81)	461 (77)	191 (90)	58 (94)
WBC	N=872	N=598	N=212	N=62
<10 x 10^9^/l	675 (77)	436 (73)	187 (88)	52 (84)
≥10 x 10^9^/l	197 (23)	162 (27)	25 (12)	10 (16)
Platelet count	*N*=871	*N*=598	*N*=212	*N*=61
<400 x 10^9^/l	779 (89)	524 (88)	200 (94)	55 (90)
≥400 x 10^9^/l)	92 (11)	74 (12)	12 (6)	6 (10)
NLR	*N*=871	*N*=597	*N*=212	*N*=62
<3	325 (37)	198 (33)	95 (45)	32 (52)
≥3	546 (63)	399 (67)	117 (55)	30 (48)
<5	617 (71)	383 (64)	177 (83)	57 (92)
≥5	254 (29)	214 (36)	35 (17)	5 (8)
Albumin	*N*=847	*N*=582	*N*=205	*N*=60
<35 g/l	210 (25)	177 (30)	26 (13)	7 (12)
≥35 g/l	637 (75)	405 (70)	179 (87)	53 (88)
Total Bilirubin	*N*=869	*N*=597	*N*=210	*N*=62
Grade 0	791 (91)	534 (89)	197 (94)	60 (97)
Grade ≥1	78 (9)	63 (11)	13 (6)	2 (3)
AST	*N*=868	*N*=596	*N*=212	*N*=60
Grade 0	564 (65)	344 (58)	165 (78)	55 (92)
Grade ≥1	304 (35)	252 (42)	47 (22)	5 (8)
ALT	*N*=871	*N*=596	*N*=213	*N*=62
Grade 0	715 (82)	471 (79)	187 (88)	57 (92)
Grade ≥1	156 (18)	125 (21)	26 (12)	5 (8)
ALP	*N*=854	*N*=587	*N*=205	*N*=62
Grade 0	353 (41)	201 (34)	109 (53)	43 (69)
Grade ≥1	501 (59)	386 (66)	96 (47)	19 (31)
Primary tumour site				
Right colon	244 (27)	176 (28)	51 (23)	17 (27)
Left colon	593 (65)	405 (64)	149 (68)	39 (62)
Not specified	76 (8)	50 (8)	19 (9)	7 (11)
Time from first metastasis to first FTD/TPI intake^c^				
<18 months	161 (18)	129 (20)	28 (13)	4 (6)
≥18 months	752 (82)	503 (80)	190 (87)	59 (94)
No. metastatic sites^d^				
1-2	734 (81)	496 (79)	184 (84)	54 (86)
≥3	177 (19)	134 (21)	34 (16)	9 (14)
Liver metastasis	670 (73)	486 (77)	147 (67)	37 (59)
Lung metastasis	426 (47)	280 (44)	115 (53)	31 (49)
Lymph node metastasis	208 (23)	143 (23)	49 (22)	16 (25)
*RAS* mutation-positive^e^	438 (53)	326 (56)	88 (46)	24 (42)
Previous treatment containing anti-VEGF	784 (86)	547 (87)	185 (84)	52 (83)
Previous treatment containing regorafenib	283 (31)	207 (33)	62 (28)	14 (22)

^a^n (%) unless otherwise stated. ^b^27 patients did not have ECOG PS data available. ^c^One patient did not have time from first metastasis to first FTD/TPI intake measurable. ^d^Three patients with missing data. ^e^186 patients did not have a RAS status evaluable. ALT, alanine aminotransferase; ALP, alkaline phosphatase; AST, aspartate aminotransferase; DoT, duration of treatment. ECOG PS, Eastern Cooperative Oncology Group performance status. FTD/TPI, trifluridine/tipiracil. Min, minimum. Max, maximum, NLR, neutrophil:lymphocyte ratio; WBC, white blood cells

### Safety

TEAEs of any grade were experienced by 96.6% of the overall patient population (Table 2). The frequency of severe (grade ≥ 3) TEAEs increased with DoT (Table 2). The most frequent severe TEAEs were neutropenia, anaemia, asthenia, and diarrhoea (Table 2). Severe febrile neutropenia (1.2%) and severe cardiac disorders (0.7%) were infrequent, and this frequency was not related to DoT.

**Table 2:** Summary of safety in the whole population in PRECONNECT and by duration of FTD/TPI treatment

	Frequency in each population, n (%)			
TEAE, n (%)	All	0–3 cycles	4–7 cycles	≥8 cycles
	*N* = 914	*n* = 632	*n* = 219	*n* = 63
Any TEAE	883 (96.6)	611 (96.7)	209 (95.4)	63 (100.0)
Severe TEAE (grade ≥ 3)	683 (74.7)	466 (73.7)	160 (73.1)	57 (90.5)
Most frequent severe TEAE (grade ≥ 3)				
Neutropenia^a^	355 (38.8)	180 (28.5)	127 (58.0)	48 (76.2)
Anaemia^a^	103 (11.3)	65 (10.3)	30 (13.7)	8 (12.7)
Asthenia^a^	49 (5.4)	36 (5.7)	10 (4.6)	3 (4.8)
Diarrhoea	39 (4.3)	27 (4.3)	8 (3.7)	4 (6.3)
Severe TEAE (grade ≥ 3) of interest				
Febrile neutropenia^a^	11 (1.2)	8 (1.3)	2 (0.9)	1 (1.6)
Cardiac disorders^a^	6 (0.7)	5 (0.8)	1 (0.5)	–
TEAE related to study drug	721 (78.9)	461 (72.9)	199 (90.9)	61 (96.8)
Severe TEAE (grade ≥ 3) related to study drug	459 (50.2)	262 (41.5)	144 (65.8)	53 (84.1)
Most frequent severe TEAE (grade ≥ 3) related to study drug				
Neutropenia^a^	348 (38.1)	175 (27.7)	126 (57.5)	47 (74.6)
Anaemia^a^	66 (7.2)	37 (5.9)	23 (10.5)	6 (9.5)
Asthenia^a^	31 (3.4)	21 (3.3)	8 (3.7)	2 (3.2)
Diarrhoea	28 (3.1)	21 (3.3)	5 (2.3)	2 (3.2)
Severe TEAE (grade ≥ 3) of interest related to study drug				
Febrile neutropenia^a^	11 (1.2)	8 (1.3)	2 (0.9)	1 (1.6)
Cardiac disorders^a^	1 (0.1)	1 (0.2)	–	–
TEAE leading to study drug withdrawal	195 (21.3)	169 (26.7)	20 (9.1)	6 (9.5)
TEAE leading to study drug dose reduction	106 (11.6)	57 (9.0)	36 (16.4)	13 (20.6)
Study drug-related	93 (10.2)	48 (7.6)	32 (14.6)	13 (20.6)
TEAE leading to study drug delay or temporary interruption	481 (52.5)	285 (44.9)	145 (66.2)	51 (81.0)
Study drug-related	384 (42.0)	206 (32.6)	131 (59.8)	47 (74.6)

^a^Analysed per group term. TEAE, treatment-emergent adverse event

The most frequent drug-related severe TEAEs were neutropenia (38.1%), anaemia (7.2%), and asthenia (3.4%) (Table 2). Drug withdrawal due to TEAEs decreased with longer DoT while both study drug dose reduction and delay or temporary interruption of study drug due to TEAEs increased with DoT (Table 2). A serious TEAE was reported by 335 (36.7%) patients of which 97 patients (10.6%) had at least one serious TEAE related to study drug. Serious TEAEs occurred in 263 (41.6%), 52 (23.7%) and 20 (31.7%) patients receiving 0–3 cycles, 4–7 cycles and ≥ 8 cycles of FTD/TPI, respectively.

**Fig. 1:**
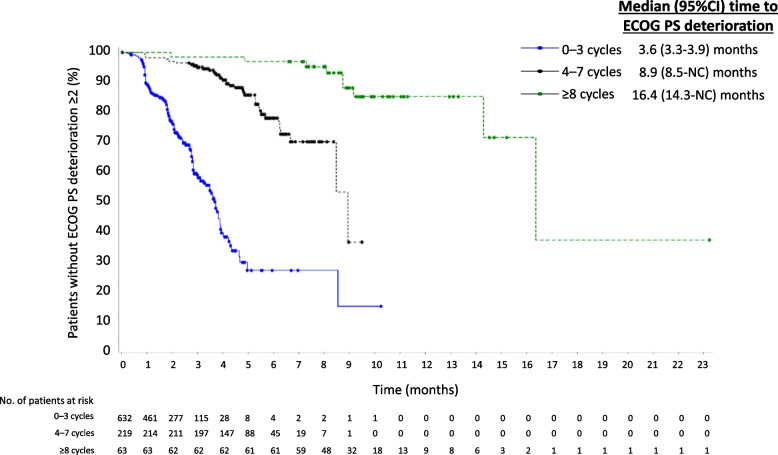
Median time to ECOG PS deterioration by duration of FTD/TPI treatment. CI, confidence interval. ECOG PS, Eastern Cooperative Oncology Group performance status. FTD/TPI, trifluridine/tipiracil. NC, not calculable

Median (95% CI) time to ECOG PS deterioration to ≥2 was 8.7 (8.1–NC) months in the overall population. Time to ECOG PS deterioration to ≥2 increased with DoT from a median of 3.6 (3.3–3.9) months in the group completing 0–3 cycles to 16.4 (14.3–NC) months in the group completing ≥8 cycles (Fig. 1).

### Efficacy

**Fig. 2:**
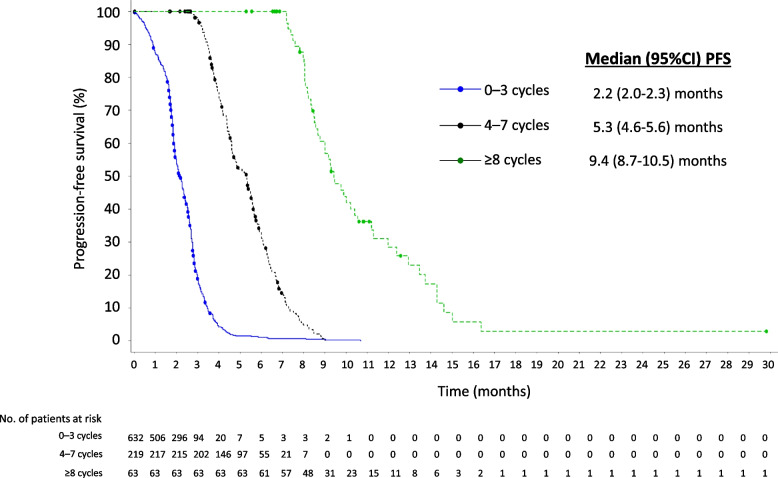
Median progression-free survival by duration of FTD/TPI treatment. CI, confidence interval. FTD/TPI, trifluridine/tipiracil. PFS, progression-free survival

FTD/TPI treatment was associated with a median (95% CI) PFS of 2.8 (2.7–3.0) months in the overall population. Median PFS increased with increasing durations of FTD/TPI treatment, from 2.2 (2.0–2.3) months in the group completing 0–3 cycles to 9.4 (8.7–10.5) months in the group completing ≥8 cycles (Fig. 2).

ORR was 2.1% (95% CI 1.3–3.2) in the PRECONNECT population, while DCR was 33.3% (95% CI 30.2–36.4). ORR increased with longer durations of FTD/TPI treatment: 0.6% (95% CI 0.2–1.6), 4.6% (95% CI 2.2–8.2) and 7.9% (95% CI 2.6–17.6) with 0–3, 4–7 and ≥ 8 cycles, respectively. DCR increased with longer durations of FTD/TPI treatment: 12.3% (95% CI 9.9–15.2), 75.3% (95% CI 69.1–80.9), and 96.8% (95% CI 89.0–99.6), with 0–3, 4–7 and ≥ 8 cycles, respectively.

### Exploratory analyses of prognostic factors

Baseline characteristics associated with neutropenia and PFS were explored in the analysis of potential prognostic factors. In total, 836 patients with complete data on prognostic factors were retained in the final model of prognostic factors for neutropenia or severe neutropenia, and 808 patients were retained in the final model to investigate potential prognostic factors for PFS.

Identified prognostic factors favouring the occurrence of neutropenia during treatment with FTD/TPI were time since diagnosis of the first metastasis of ≥18 months, baseline haemoglobin ≥110 g/l, NLR < 3, baseline AST grade 0, baseline albumin ≥35 g/l, previous lines of treatment > 2, and baseline white blood cell count < 10 × 10^9^/l (Supplementary Table 2). Identified prognostic factors favouring the occurrence of severe neutropenia during treatment with FTD/TPI were time since diagnosis of the first metastasis of ≥18 months, absence of liver metastasis, age ≥ 70 years, presence of previous anti-VEGF treatment, previous lines of treatment > 2, baseline NLR < 5, baseline AST grade 0, baseline albumin ≥35 g/l, and baseline white blood cell count < 10 × 10^9^/l (Supplementary Table 2).

**Fig. 3:**
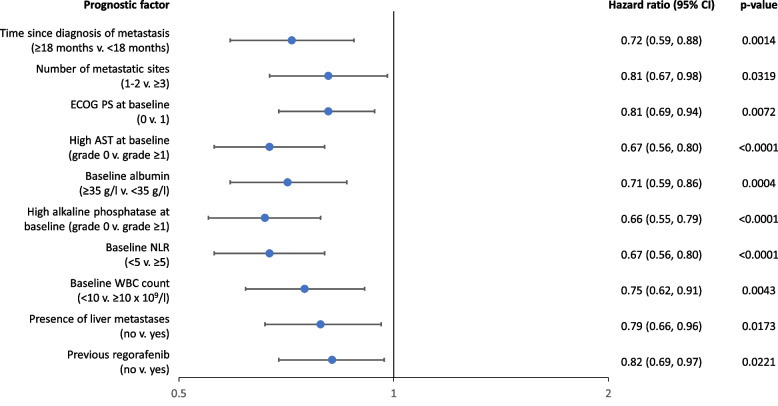
Prognostic factors for PFS (*n* = 808). AST, aspartate aminotransferase. CI, confidence interval. ECOG PS, Eastern Cooperative Oncology Group performance status. NLR, neutrophil-lymphocyte ratio. PFS, progression-free survival. WBC, white blood cell count

Identified prognostic factors associated with longer PFS were time since diagnosis of the first metastasis of ≥18 months, < 3 metastatic sites, ECOG-PS 0 at baseline, absence of liver metastasis, no previous treatment with regorafenib, baseline NLR < 5, baseline alkaline phosphatase grade 0, baseline AST grade 0, baseline albumin ≥35 g/l, and baseline white blood cell count < 10 × 10^9^/l (Fig. 3).

Median PFS was longer in patients experiencing neutropenia during FTD/TPI treatment compared with those having no neutropenia (Supplementary Fig. 1A), and in patients with severe neutropenia during treatment compared with patients with no severe neutropenia (Supplementary Fig. 1B). Cox regression analyses indicated that there was a significant association between PFS and neutropenia occurrence over time (hazard ratio [HR]: 0.36 [95% CI: 0.31, 0.42]) or severe neutropenia over time (HR: 0.44 [95% CI: 0.38, 0.51]).

### Quality of life

QoL was maintained during treatment with FTD/TPI in the overall population and in the DoT subgroups: there were no clinically relevant changes (> 10-point mean change from baseline) in QLQ-C30-GHS scores during the study (Supplementary Fig. 2A and B). Non-censored data for time to global health deterioration (*n* = 422) demonstrated that 387 patients had a ≥ 10 point definitive deterioration and 35 patients died. Median time to deterioration of global health status was 3.7 months (95% CI: 3.3, 3.8).

## Discussion

The final results from the PRECONNECT study in patients with mCRC in daily clinical practice confirm the interim data published in 2020 [11], that showed consistency with the safety profile and PFS that was previously reported in the phase III RECOURSE study [5]. The safety profile of FTD/TPI and maintenance of QoL (discussed in more detail in the publication of interim results) [11] were consistent regardless of DoT. Disease progression was the most frequent reason for withdrawal from PRECONNECT, and toxicity and ECOG PS deterioration rarely led to withdrawal. The DoT data suggest that FTD/TPI can be used in multiple cycles with confidence in routine practice.

There is a lack of information on potential prognostic factors in later lines of treatment in patients with mCRC. In patients naïve to chemotherapy treatment, CEA, number of metastatic sites, age, NLR, lactate dehydrogenase levels, and primary tumour location, have been shown to be important prognostic indicators [15–18]. However, as later lines of treatment for mCRC have only emerged in the last 10 years, prognostic factors in patients already heavily pre-treated, have not been extensively studied. In addition, prognostic factors derived from patients enrolled in clinical trials may not reflect those observed in daily clinical practice. The PRECONNECT study included the largest series of patients receiving FTD/TPI in real-world practice following ≥2 previous lines of treatment, and therefore provides an opportunity to investigate relevant prognostic factors linked to disease progression or the main treatment related toxicity (i.e., neutropenia). The findings of PRECONNECT show that time since diagnosis of the first metastasis and number of metastatic sites were significant prognostic values for PFS in patients treated with FTD/TPI; an observation that is largely consistent with the ad hoc analysis performed with data from RECOURSE [7]. Additional prognostic factors for PFS included ECOG-PS, absence of liver metastases, and baseline laboratory values for NLR, alkaline phosphatase, AST, albumin and white blood cell count.

In addition, median PFS was longer in patients experiencing neutropenia or severe neutropenia during FTD/TPI treatment (and importantly, most neutropenia occurred during the first three cycles of FTD/TPI treatment and this frequency during the first three cycles was higher in patients who went on to have a longer DoT [data not shown]). These results were expected as higher exposure of patients with mCRC increases the risk of neutropenia, and hence, patients developing neutropenia have improved OS and PFS [19]. Interestingly, neutropenia occurrence also appears to be a predictor of prolonged OS and PFS when FTD/TPI is combined with bevacizumab [20, 21]. The PRECONNECT study not only confirms neutropenia as a predictor of response in a large population and in routine practice, but also provides information on prognostic factors for neutropenia and severe neutropenia that may help guide individualised treatment in future (i.e. including the use of granulocyte-colony stimulating factor [G-CSF]). Meta-analyses of data from randomised controlled trials suggest that G-CSF may be used when neutropenia occurs to maintain the dose-intensity of cytotoxic treatment and improve OS [22, 23]. A phase II trial in patients with grade 3–4 neutropenia is currently underway to investigate the efficacy of secondary prophylaxis with G-CSF in the maintenance of FTD/TPI dose intensity and its effect on FTD/TPI treatment efficacy (https://clinicaltrials.gov/ct2/show/NCT04166604).

Limitations of PRECONNECT include the lack of a comparator arm and the lack of follow-up data which meant that OS was not assessed. One of the reasons OS could not be assessed was the withdrawal criterion of FTD/TPI commercial availability, which accounted for 10% of all withdrawals. However, this is unlikely to have an impact on the other conclusions from the study due to the relatively large study population recruited from 16 countries, which helps to define the safety of FTD/TPI more broadly than is possible with controlled clinical trials. As PRECONNECT was a ‘real-world’ study, follow-up assessments were based on the standard of care at each centre; this meant that, for example, the date of assessment of response may have varied in the recruited population (e.g., it could be 2 months at one centre and 3 months at another), which would have an impact on the PFS outcomes.

In summary, findings from the PRECONNECT study (which, with 914 patients, constitutes the largest real-world cohort of patients receiving FTD/TPI to date) lend additional support and confidence for the routine use of FTD/TPI, including the use of multiple cycles, for the treatment of patients with mCRC in daily clinical practice. The results demonstrate that neutropenia is a potential predictor of response in the real-world setting. Median time to ECOG PS deterioration was 8.7 months, median PFS was 2.8 months and both increased with duration of treatment. Our understanding of the efficacy and safety of FTD/TPI stem largely from the pivotal RECOURSE clinical trial – PRECONNECT enhances our understanding of the safety and efficacy in the real-world and when FTD/TPI is used over longer durations.

## Supplementary Information

**Additional file 1.**

## Data Availability

Data are available on reasonable request. All data relevant to the study are included in the article or uploaded as supplementary information. Anonymised patient-level, study-level clinical trial data will be shared in agreement with the Servier Data-Sharing Policy available at https://clinicaltrials.servier.com/data-request-portal/.
