# Supplementary material for: Safety and efficacy of trifluridine/tipiracil in previously treated metastatic colorectal cancer: final results from the phase IIIb single-arm PRECONNECT study by duration of therapy
Source: BMC Cancer. 2023 Jan 27;23:94. doi: 10.1186/s12885-022-10489-4 (PMC9881327; doi:10.1186/s12885-022-10489-4)
Supplement: Supplementary file 1 — Additional file 1. [file 12885_2022_10489_MOESM1_ESM.docx]

**Supplementary tables and figures**

Supplementary Table 1. FTD/TPI relative dose intensity

| Dose intensity, n (%) | All N=911^a^ | 0–3 cycles  n=630 | 4–7 cycles  n=218 | ≥8 cycles  n=63 |
| --- | --- | --- | --- | --- |
| ≤60 | 32 (3.5) | 30 (4.8) | 1 (0.5) | 1 (1.6) |
| >60 – ≤80 | 169 (18.6) | 101 (16.0) | 49 (22.5) | 19 (30.2) |
| >80 – ≤100 | 630 (69.2) | 432 (68.6) | 157 (72.0) | 41 (65.1) |
| >100 – ≤110 | 61 (6.7) | 48 (7.6) | 11 (5.1) | 2 (3.2) |
| >110 | 19 (2.1) | 19 (3.0) | – | – |
| Median (min, max) | 90.3 (19.9, 239.7) | 92.8 (19.9, 239.7) | 86.2 (57.3, 102.5) | 83.3 (55.1, 100.4) |

^a^ Three patients with missing data for dose intensity

Max, maximum. Min, minimum

**Supplementary Table 2.** Prognostic factors for the occurrence of neutropenia or severe neutropenia during treatment with FTD/TPI (n=836)

| Prognostic factor | Odds ratio (95% CI) | p-value |
| --- | --- | --- |
| **Neutropenia** |  |  |
| Time since first metastasis (≥18 months v. <18 months) | 1.87 (1.23, 2.84) | 0.0036 |
| Baseline haemoglobin (≥110 v. <110 g/l) | 2.21 (1.44, 3.339) | 0.0003 |
| Baseline WBC (<10 vs. ≥10 x10^9^/l) | 5.21 (3.42, 7.94) | <0.0001 |
| Baseline NLR (<3 v. ≥3) | 1.63 (1.16, 2.28) | 0.0044 |
| Baseline albumin (≥35 v. <35 g/l) | 2.36 (1.60, 3.49) | <0.0001 |
| Baseline AST (grade 0 v. grade ≥1) | 1.81 (1.30, 2.52) | 0.0004 |
| Number of previous treatment lines  (≤2 v. >2) | 0.69 (0.50, 0.96) | 0.0283 |
| **Severe neutropenia** |  |  |
| Time since first metastasis (≥18 months v. <18 months) | 1.81 (1.16, 2.80) | 0.0084 |
| Presence of liver metastasis (no v. yes) | 1.36 (0.96, 1.92) | 0.0801 |
| Age (<70 v. ≥70 years) | 0.61 (0.42, 0.87) | 0.0064 |
| Presence of previous anti-VEGF treatment (no v. yes) | 0.70 (0.48, 1.04) | 0.0798 |
| Baseline WBC (<10 vs. ≥10 x10^9^/l) | 4.09 (2.55, 6.55) | <0.0001 |
| Baseline NLR (<5 v. ≥5) | 1.86 (1.27, 2.72) | 0.0015 |
| Baseline albumin (≥35 v. <35 g/l) | 1.69 (1.13, 2.53) | 0.0103 |
| Baseline AST (grade 0 v. grade ≥1) | 1.65 (1.17, 2.32) | 0.0046 |
| Number of previous treatment lines  (≤2 v. >2) | 0.70 (0.51, 0.97) | 0.0317 |

AST, aspartate aminotransferase. CI, confidence interval. NLR, neutrophil-lymphocyte ratio. WBC, white blood cell count.

**Supplementary Table 3.** Ethics Committees who approved the study in each country

| **COUNTRY** | **Names of the Ethics Committee that approved the study** |
| --- | --- |
| AUSTRALIA | Central Adelaide Local Health Network (CALHN) The Queen Elizabeth Hospital Human Research Ethics Committee (TQEH/LMH/MH) The Queen Elizabeth Hospital Basil Hetzel Institute DX465101 28 Woodville Road Woodville South SA 5011 Australia  Bellberry Human Research Ethics Committee 129 Glen Osmond Road EASTWOOD SOUTH AUSTRALIA 5063 Australia  Cabrini Health Human Research Ethics Committee Cabrini Education and Research Precinct 154 Wattletree Road MALVERN VICTORIA 3144 Australia |
| BELGIUM | Commissie Medische Ethiek UZ / KU Leuven Herestraat 49 3000 Leuven |
| BRAZIL | Comitê de Ética em Pesquisa do Hospital Mãe de Deus/Associação Educadora São Carlos - AESC Comitê de Ética em Pesquisa em Seres Humanos da Fundação Bahiana de Cardiologia Comitê de Ética em Pesquisa da Fundação Antonio Prudente – AC Camargo Câncer Center Comitê de Ética em Pesquisa do Instituto Brasileiro de Controle do Câncer IBCC Comitê de Ética em Pesquisa do Hospital Pró-cardíaco Comitê de Ética em Pesquisa da USP - Faculdade de Medicina da Universidade de São Paulo - FMUSP |
| BULGARIA | Ethics Committee for multicentre trials 5 Sveta Nedelia squarre SOFIA 1000 |
| CROATIA | Sredisnje eticko povjerenstvo Agencija za lijekove i medicinske proizvode Ksaverska cesta 4, 10000 Zagreb Republika Hrvatska |
| FRANCE | CPP Sud Méditerranée 1 Hopital Sainte Marguerite  Batiment Direction  270, Bd Sainte Marguerite  13274 Marseille Cedex 9 |
| IRELAND | Clinical Research Ethics Committee of the Cork Teaching Hospital Secretariat Lancaster Hall 6 Little Hanover Street Cork |
| ITALY | Gemelli - Fondazione Policlinico Universitario "Agostino Gemelli" Università Cattolica del Sacro Cuore  Largo Agostino Gemelli 8 - 00168 ROMA  Comitato Etico dell’Azienda Ospedaliera Arcispedale Santa Maria Nuova/I.R.C.C.S. – c/o Infrastruttura Ricerca-Statistica - Edificio Spallanzani – Viale Umberto I, 50 - 42123 REGGIO EMILIA  COMITATO ETICO PALERMO 2  c/o Azienda Ospedaliera Ospedali Riuniti  Villa Sofia - Cervello Viale Strasburgo, 233 - 90146 PALERMO  COMITATO ETICO CATANIA 2 Azienda Ospedaliera di Rilievo Nazionale e di Alta Specializzazione Garibaldi Piazza S.M. di Gesù, 7 - 95122 CATANIA  Comitato Etico Seconda Università degli Studi di Napoli (A.O.U. S.U.N.) - AORN Ospedale dei Colli Via Santa Maria di Costantinopoli, 104  80138 NAPOLI  COMITATO ETICO CAMPANIA NORD per la sperimentazione e ricerca biomedica AZIENDA OSPEDALIERA “SAN GIUSEPPE MOSCATI” DI AVELLINO Contrada Amoretta – Città Ospedaliera - Pal. Uffici 83100 AVELLINO  Comitato Etico Indipendente Fondazione IRCCS Istituto Nazionale dei Tumori Via Giacomo Venezian, 1 20133 MILANO  COMITATO ETICO REGIONE TOSCANA - AREA VASTA CENTRO CEAVCE Azienda Ospedaliero Universitaria CareggiPad. 3 - NIC - DidatticaLargo Brambilla, 3 - 50134 FIRENZE  COMITATO ETICO REGIONE TOSCANA - AREA VASTA NORD-OVEST - CEAVNO Sezione Autonoma del Comitato Etico Regionale per la sperimentazione Clinica Azienda Ospedaliero-Universitaria Pisana Via Roma, 67 - 56126 PISA  Segreteria Tecnico-Scientifica Centrale Comitato Etico IRST IRCCS AVR c/o IRST IRCCS Via P. Maroncelli, 40 – 47014 Meldola (FC)  REGIONE ABRUZZO COMITATO ETICO PER LA RICERCA BIOMEDICA DELLE PROVINCE DI CHIETI E PESCARA Via Dei Vestini 29/B – 66100 CHIETI  Comitato Etico Istituto Tumori "Giovanni Paolo II"IRCCS - Ospedale Oncologico di Bari Viale Orazio Flacco, 65 - 70124 BARI  Comitato Etico Istituto Oncologico Veneto - I.R.C.C.S. c/o Palazzo Santo Stefano Piazza Antenore,3 - 35121 PADOVA  Sezione del CE IRCCS Istituto Tumori "Giovanni Paolo II" di Bari C/O FONDAZIONE CASA SOLLIEVO DELLA SOFFERENZA - V.le Cappuccini 1 - 71013 San Giovanni Rotondo (FG)  Comitato Etico Interaziendale A.O.U. Città della Salute e della Scienza di Torino - A.O. Ordine Mauriziano - A.S.L. TO1 Corso Bramante 88/90 - 10126 TORINO  Comitato Etico Provinciale Provincia di Brescia A.O. Spedali Civili di Brescia Piazzale Spedali Civili, 1 25123 BRESCIA  Comitato Etico Indipendente IRCCS Istituto Clinico HUMANITAS Via A. Manzoni 56 - 20089 Rozzano (MI)  COMITATO ETICO DELL'UNIVERSITA' CAMPUS BIO MEDICO DI ROMA Via Alvaro del Portillo, 200 - 00128 ROMA |
| PANAMA | Comité de Bioética de la Investigación del Instituto Conmemorativo Gorgas de Estudios de la Salud (ICGES) |
| POLAND | Komisja Bioetyczna przy Centrum Onkologii – Instytucie im. Marii Sklodowskiej-Curie w Warszawie ul. Roentgena 5 02-781 Warszawa |
| PORTUGAL | Comissão de Ética para a Investigação Clínica Parque da Saúde de Lisboa Av. do Brasil 53 – Pav. 17A 1749-004 Lisboa |
| ROMANIA | Comisia Națională de Bioetică a Medicamentului și Dispozitivelor Medicale Str. Ștefan cel Mare, Nr. 19-21 020125, București |
| SLOVAKIA | Central Ethics Committee: Eticka Komisia  Narodny Onkologicky Ustav Klenova 1 833 10 BRATISLAVA Slovenska Republika  Etická komisia Národný Onkologický Ústav Klenová 1 833 10 Bratislava  Etická komisia Onkologický ústav sv. Alžbety, s.r.o.  Heydukova 10 812 50 Bratislava  Etická komisia Východoslovenský onkologický ústav, a.s. Rastislavova 43 041 91 Košice  Etická komisia  Univerzitná nemocnica Martin Kollárova 2 036 01 Martin |
| SLOVENIA | Komisija RS za medicinsko etiko Ministrstvo za zdravje Štefanova 5 1000 Ljubljana |
| TURKEY | Gazi University Medical Faculty Ethics Committee 06500 Teknikokullar - ANKARA |
| UKRAINE | Ethics Committee at Kyiv City Clinical Cancer Center, Verkhovynnaya str., 69, 03115, Kiev, Ukraine;   Ethics Committee at Treatment-diagnostic Centre PJSC “House of medicine”, 69/17 Rozkydaylivska str, Odessa, 65000, Ukraine;   Ethics Committee at Clinical and diagnostic Centre of “Medics-rey International Group LLC”, Hospital of Israeli Oncology “LISOD”, 27 A. Malyshko str. Plyuti village, 08720, Kyiv Region, Obukhiv district, Ukraine;   Ethics Committee at Kharkiv Regional Oncology Center, Lesoparkovskaya str., 4, 61070, Kharkiv, Ukraine; |

**Supplementary Figure 1.** Median progression-free survival by presence or absence of (A) neutropenia or (B) severe neutropenia during treatment

(A)


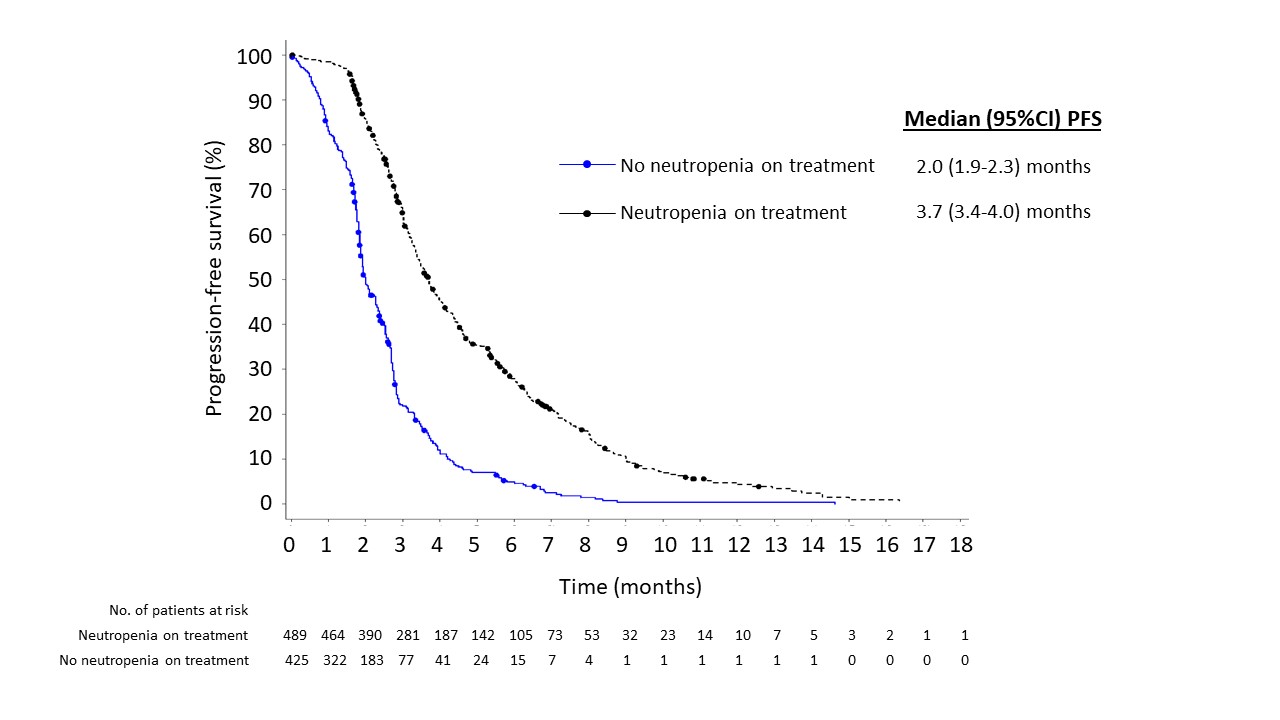


(B)


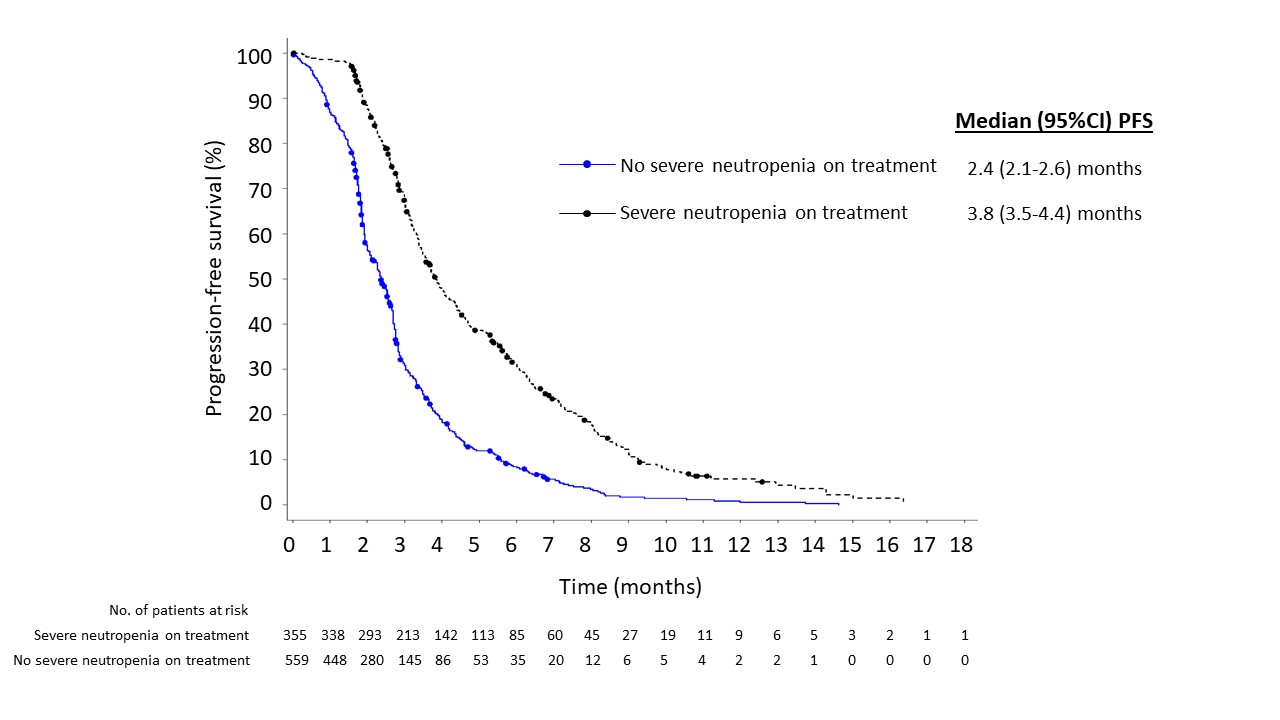


CI, confidence interval. FTD/TPI, trifluridine/tipiracil. PFS, progression-free survival.

**Supplementary Figure 2.** Evolution of global health status (QLQ-C30) in the overall population (A) and by DoT subgroup (B)*

(A)

Improvement

Deterioration

No change

754

n

516

299

202

150

89

66

46


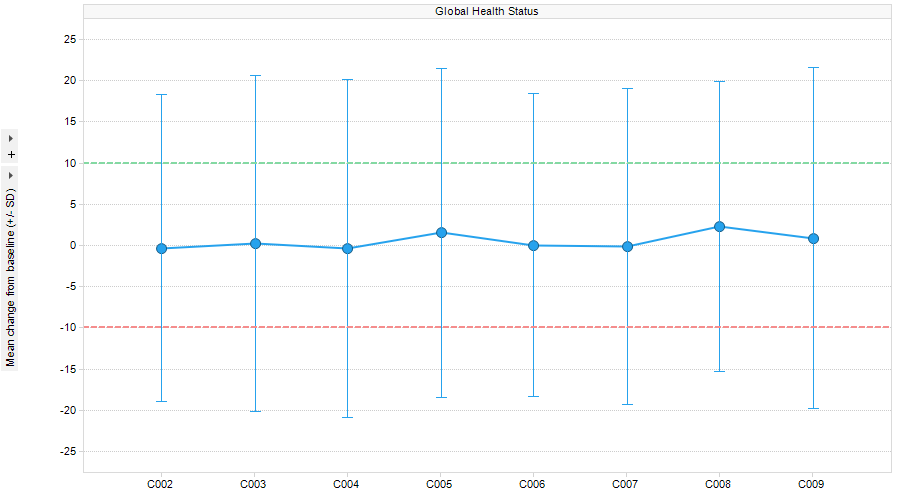


(B)

**
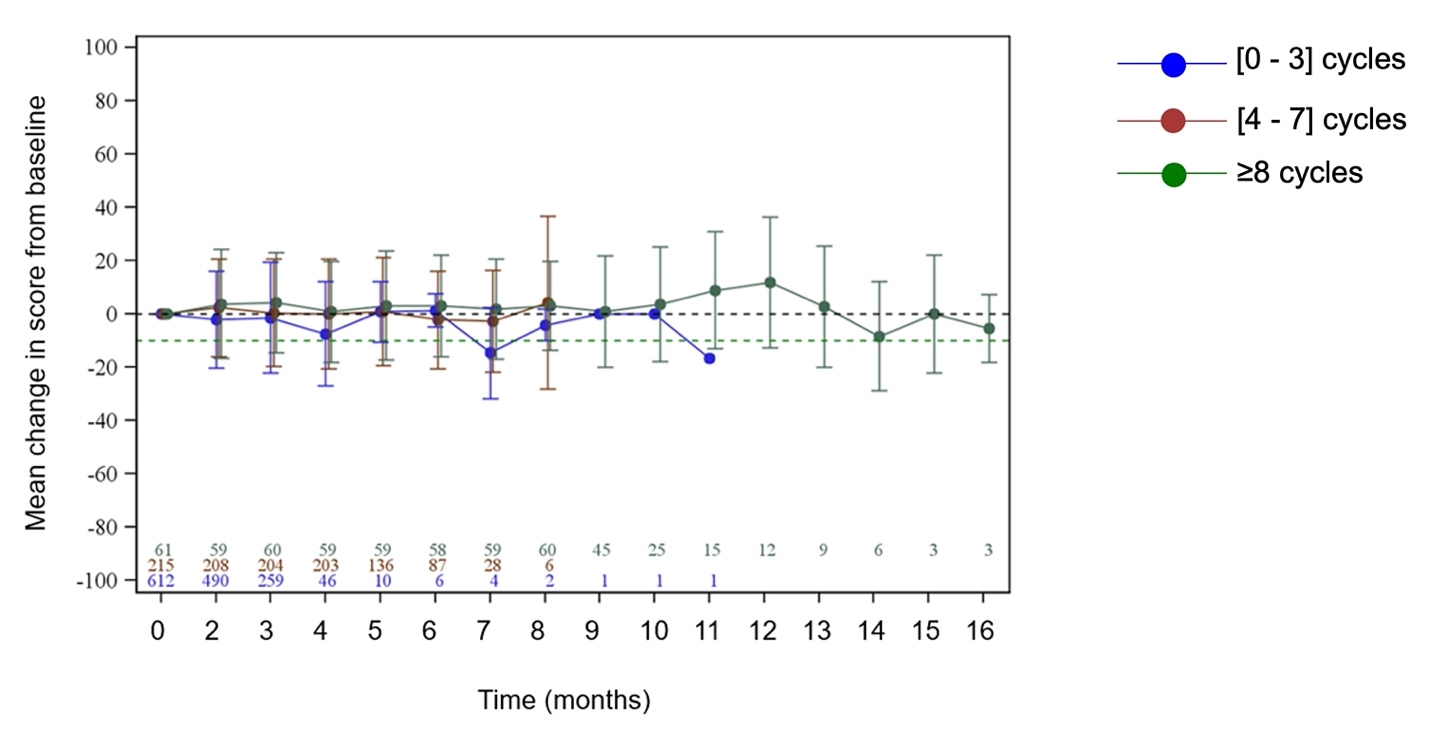
**

*Some patients in group 0–3 were followed until cycle 11 but only completed ≤3 cycles.

DoT, duration of therapy.
